# G-quadruplex structures as modulators of alternative promoter usage

**DOI:** 10.1093/nargab/lqaf208

**Published:** 2025-12-31

**Authors:** Rongxin Zhang, Jean-Louis Mergny

**Affiliations:** Laboratoire d’Optique et Biosciences (LOB), Ecole Polytechnique, CNRS, INSERM, Institut Polytechnique de Paris, Palaiseau 91120, France; State Key Laboratory of Digital Medical Engineering, School of Biological Science and Medical Engineering, Southeast University, Nanjing 211189, China; Laboratoire d’Optique et Biosciences (LOB), Ecole Polytechnique, CNRS, INSERM, Institut Polytechnique de Paris, Palaiseau 91120, France

## Abstract

The precise regulation of gene transcription relies on promoters, and the selection of specific promoters for a particular gene is a key determinant of transcript diversity. However, the regulatory mechanisms governing promoter selection are not fully understood. G-quadruplexes (G4s) are unique DNA noncanonical secondary structures that have emerged as important regulators of gene expression. In this study, we systematically analyzed the relationship between G4 structures and alternative promoters (APs) in two cancer cell lines, K562 and HepG2, by integrating native elongating transcript-cap analysis of gene expression and G4 ChIP-seq datasets. We identified 573 differentially utilized APs (|fold change| > 2, false discovery rate < 0.05), 26% of which being associated with G4 structures within 100 base pairs. Notably, G4-associated promoters predominantly exhibited increased activity, suggesting that G4s generally promote AP selection. Furthermore, treatment with G4 ligands induced the generation of APs, suggesting that the stabilization of G4 structures may modulate AP usage. Collectively, these findings provide new insights into the G4-based mechanisms that regulate transcript isoform diversity.

## Introduction

Transcription is tightly regulated by promoters, located close to the transcription start sites (TSSs). These promoters regulate gene expression by interacting with transcription factors and other regulatory elements [1]. Extensive studies have shown that numerous human genes possess more than one promoter [2, 3]. The use of different promoters, known as alternative promoters (APs), contributes to the diversity of transcripts [4, 5], which in turn modulates a wide range of gene functions [4, 5]. A previous pan-cancer study revealed that APs are common across multiple cancer types. The selective activation of APs contributes to transcript diversity, which can influence patient survival independently of overall gene expression changes between cancerous and normal tissues [6]. Coincidentally, another report demonstrated that APs may serve as novel biomarkers for assessing the prognosis of multiple myeloma patients [7].

Recent studies have identified several factors that can regulate promoter selection in cancers, including DNA methylation, chromatin accessibility, and histone modifications [8–10]. Nevertheless, our understanding of intracellular determinants influencing promoter selection remains incomplete. Therefore, identifying additional regulatory elements involved in that process is of significant value.

G-quadruplexes (G4s) are noncanonical secondary structures that typically form in G-rich regions [11]. Their distribution in the human genome is nonrandom, with a predominant enrichment in gene promoter regions, suggesting a potential regulatory role in transcription [12, 13]. To date, numerous studies have emphasized the strong association between G4 structures and gene expression, especially their connection with highly expressed genes [14–16]. However, these studies mainly concentrate on gene expression levels or overall transcript expression and overlook the contribution of specific TSSs. Notably, one study has preliminarily explored the association of G4s with APs [17]; however, it relies mainly on RNA-seq data, which introduces a degree of uncertainty regarding promoter-specific activity [17].

Unlike RNA-seq, native elongating transcript-cap analysis of gene expression (NET-CAGE) experiments provide a more precise landscape of the TSSs within cells [18], as they directly detect the 5′ ends of nascent transcripts instead of inferring them from read coverage. In this study, we used bioinformatics methods to analyze NET-CAGE sequencing data and identify potential promoter regions in the K562 and HepG2 cell lines. We then examined the association between endogenous G4 structures and promoters. To further explore whether the stability of G4 structures influences promoter selection, we analyzed the effects of G4 ligands (GSE133419), including PhenDC3, on promoters. PhenDC3 is a widely recognized G4 specific ligand known for its high binding affinity and selectivity for G4 structures over duplex and single-stranded DNAs [19]. It is often used as a reference compound for G4 studies due to its robust and selective binding properties.

## Materials and methods

### G-quadruplex predicted sequences and ChIP-seq data

We utilized the G4Hunter software [20, 21] to predict G4 sequences on both the positive and negative strands of the human genome (assembly version: hg19). G4Hunter is an efficient and accurate software that is designed to predict the formation propensity of G4 structures. The core principle of G4Hunter is to assign scores to consecutive guanines (G) and cytosines (C) while considering adenines (A) and thymines (T) as neutral. For example, a single G is assigned a score of +1, each G in a run of two Gs is given +2, three Gs get +3 each, while four or more consecutive Gs are all assigned a score of +4. Cytosine (C) bases are scored similarly but are given negative values. A higher absolute value of the score generally suggests a higher propensity to form G4 structures. Please note that a negative score indicates a C-rich sequence, which suggests G4 formation would occur on the complementary strand. Predictions were performed with a window size of 25 bp (default value) and a relatively conservative prediction threshold score of 1.5 (the default threshold is 1.2; a threshold of 1.5 ensures >98% confidence that the candidate sequence forms a G4 *in vitro*). Applying these criteria, we obtained a total of 1427 546 predicted G4 sequences.

We selected the K562 and HepG2 cell lines as our study subjects since their G4 profiles have been thoroughly characterized, thereby ensuring the reliability of our comparative analyses. The G4 ChIP-seq data for the K562 and HepG2 cell lines were acquired from the Gene Expression Omnibus (GEO) database (accession number: GSE145090). In this technique, the G4-specific antibody BG4 is used in a chromatin immunoprecipitation framework to selectively enrich G4-containing chromatin, and the enriched DNA fragments are subsequently sequenced to generate genome-wide G4 profiles. To further improve reliability, we used the peak data with higher reproducibility (GSE145090_20 180 108_K562_async_rep1-3.mult.5of8.bed.gz for the K562 cell line and GSE145090_HepG2_async_rep1-3.mult.6of9.bed.gz for the HepG2 cell line).

In addition, the raw G4 ChIP-seq reads were reprocessed to compute G4 signal intensity using the rtracklayer package, complementing the peak-based analyses. Specifically, the FASTQ files for the K562 and HepG2 cell lines were downloaded from the GEO database under accession numbers GSE107690 and GSE145090, respectively. The quality of the sequencing reads derived from G4 ChIP-seq was assessed using FastQC (version 0.12.0). Based on the FastQC reports, the first 15 bases of each read were removed using Trimmomatic (version 0.39). Subsequently, we aligned the quality-controlled reads to the reference genome using Bowtie2 (version 2.2.5). The resulting alignment files underwent format conversion (SAM to BAM), sorting, and the retention of uniquely mapped reads through the use of SAMtools (version 1.6) and Sambamba (version 0.6.6). We used BEDTools (version 2.30.0) to eliminate the reads that mapped to “blacklisted” regions, which were obtained from the following link: https://github.com/Boyle-Lab/Blacklist/tree/master/lists. To generate the integrated signal files, we merged the BAM files using SAMtools. Finally, we employed the built-in program bamCoverage in deepTools (version 3.5.6) to convert the BAM files into the BigWig format.

In this study, the 22 autosomes and the X chromosome were considered and retained for subsequent analysis (i.e. the Y chromosome was excluded). Unless specified otherwise, all human genome data used in this study are based on the assembly version of hg19.

### NET-CAGE datasets, tag cluster, and annotation

The publicly available processed NET-CAGE transcription start site (CTSS) data for the K562 and HepG2 cell lines were directly retrieved from GSE118075 [18], including six replicates for each cell line. NET-CAGE is a robust method that precisely maps the 5′ ends of native elongating transcripts, which correspond to the initial TSSs.

We utilized the R package CAGEfightR (version 1.18.0) [22] to identify tag clusters (TCs) in the K562 and HepG2 cell lines. This package provides a comprehensive set of tools for the analysis of 5′ end data (e.g. CAGE data), including the identification, annotation, and quantification of TSSs and enhancers. First, we converted the CTSS files into BigWig format and quantified CTSS counts and activity levels using the *quantifyCTSSs* function. Subsequently, TCs were identified from the quantified CTSS using the *quickTSSs* function. This function first calculates the tags-per-million (TPM) values and pools signals across all samples. It then merges TCs located within 20 bp (default setting) and requantifies the expression of each merged TC. TCs with activity levels >1 TPM in at least six samples (half of the total samples) were retained. Additional filtering of the TC data was performed. Candidate TCs overlapping with potential enhancers (identified from the CTSS object using the *quickEnhancers* function) were removed. Furthermore, since this study does not focus on *de novo* promoters, we also excluded TCs that did not overlap with the TSSs. TSS annotations were obtained from GENCODE release v19 (gencode.v19.annotation.gtf, *Homo sapiens*, available at https://www.gencodegenes.org/human/release_19.html) and converted into a TxDb object using the *makeTxDbFromGFF* function. We determined overlaps using the *findOverlaps* function and retained TCs that shared at least one base pair with annotated TSS. Gene annotation was performed by assigning Ensembl gene IDs to TCs with the *assignGeneID* function based on the TxDb object. To standardize identifiers, version numbers were removed from Ensembl IDs, and HGNC gene symbols were subsequently mapped using the *mapIds* function with the org.Hs.eg.db database. TCs lacking either a valid Ensembl gene ID or mapped gene symbol were discarded.

A quantitative filter was applied using the *subsetByComposition* function, retaining only TCs whose activity contributed >10% of the total promoter activity for the corresponding gene in at least six samples. Finally, only TCs corresponding to protein-coding genes on chromosomes 1–22 and X, as determined from org.Hs.eg.db, were retained for subsequent association analysis with G4 structures.

### Identification of alternative promoters based on NET-CAGE data

We followed the CAGEWorkflow (version 1.14.0) in Bioconductor to identify APs at the gene level. In this workflow tutorial, the authors used the edgeR package to identify APs, given the similarities between AP detection and alternative splicing analysis.

Promoter-level counts and associated annotations were used to create a DGEList object in edgeR (version 3.40.2) and normalized using the TMM (Trimmed Mean of M-values) method. Group information was modeled in a design matrix, and dispersion was estimated before fitting quasilikelihood negative binomial generalized linear models (GLMs). Differential promoter usage was assessed with *diffSpliceDGE* at the exon level. Genes with only one expressed promoter were excluded automatically from this step. APs were defined as those with a false discovery rate (FDR) of <0.05 and an absolute fold change > 2.

### Definition of G-quadruplex-associated promoters

The definition of a G4-associated promoter is established as follows: A TC is considered G4-associated if it contains at least one G4 ChIP-seq peak within its internal region or immediately upstream (100 bp). In other words, a TC with such G4 ChIP-seq peaks would be referred to as a G4-associated TC, which can be considered as a G4-associated promoter. Although the concepts of promoter and TC are not strictly equivalent, a TC often corresponds to a promoter that regulates it. Therefore, in this study, these two terms are considered interchangeable. We will use the characteristic of TCs, including their activity levels, to represent the promoters [23].

### Relative density calculation

The R package, EnrichedHeatmap (version 1.28.1) [24], was used to compute the relative density of G4s around APs. The calculation was performed using the coverage mode and a resolution of 10 bp.

Specifically, let *M* represent the number of APs, and *N* denote the total number of windows (window width is 10 bp in this study) over which the density of G4s will be calculated upstream and downstream of each AP. For the *n*th (1 ≤ *n* ≤ *N*) window, the density of G4s ${{d}_n}$ is calculated as follows:


\begin{eqnarray*}
{{d}_n} = \frac{{\mathop \sum \nolimits_i^M {{w}_i}}}{{10 \times N}}.
\end{eqnarray*}

Here, ${{w}_i}$ denotes the total number of bases covered by the *i*th G4 in the *n*th window.

### G4 ligand RNA-seq data analysis

The RNA-seq raw sequencing data of the HT1080-ST fibrosarcoma cell line, treated with three commonly used G4 ligands (Pyridostatin = PDS, PhenDC3, and PhenDC6) and their corresponding controls, were obtained from the GEO database (accession number of GSE133419). HT1080-ST was used in this study because the dataset contains all required ligand treatments under a single experimental framework. FastQC (version 0.12.0) was first applied for the quality assessment of sequencing reads. The first 15 bases of each read (5′ end) were then removed according to FastQC’s report using fastp (version 0.23.4). STAR (version 2.7.5) was employed to build the human genome index (assembly version: hg19) based on the GENCODE release v19 annotation file (gencode.v19.annotation.gtf). Quality-controlled reads were aligned to the genome with STAR, and the output included coordinate-sorted BAM files as well as gene-level read counts (–quantMode TranscriptomeSAM GeneCounts). Splice junction information (SJ.out.tab) was also produced and used for subsequent identification of APs. Additionally, the aligned BAM files were converted into the BigWig format for visualization in genome browsers using the built-in bamCoverage tool from deepTools (version 3.5.6) with default parameters.

### Identification of alternative promoters based on G4-ligand-treated RNA-seq data

The identification of APs after G4 ligand treatment was accomplished based on proActiv (version 1.16.0) and edgeR (version 4.4.1).

The annotation file of the human genome obtained from the GENCODE database (GENCODE release v19), together with the junction files generated during the RNA-seq alignment step, were used for the identification and quantification of promoters through proActiv. Single-exon transcripts and internal promoters were excluded. Next, the promoter counts quantified by proActiv were utilized to identify the alternative promoters. Considering the limitation in sample size, and to more sensitively detect subtle changes in promoter activity following G4 ligand treatment, we chose not to use the standard proActiv workflow. Instead, we employed edgeR for the identification of APs. First, promoter-level read counts were extracted from the proActiv output and organized into a DGEList object. Promoters with low expression were removed by requiring a counts-per-million value >1 in at least one sample. We then recalculated the library sizes and used the TMM method (default method) to estimate the normalization factors. Next, we fitted a GLM with experimental conditions (e.g. ligand-treated samples versus control samples) as the design matrix, and estimated dispersions without tagwise shrinkage. A quasilikelihood F-test (glmQLFTest) was used to assess differential promoter usage within each gene. Given that the identification of APs was equivalent to the identification of alternative splicing; therefore, in this study, the diffSpliceDGE method of the edgeR software was utilized to determine the APs. The promoters with an absolute fold change > 2 and an FDR value < 0.05 were considered as APs.

## Results

### G4 structures contribute to the formation of alternative promoters

**Figure 1.**
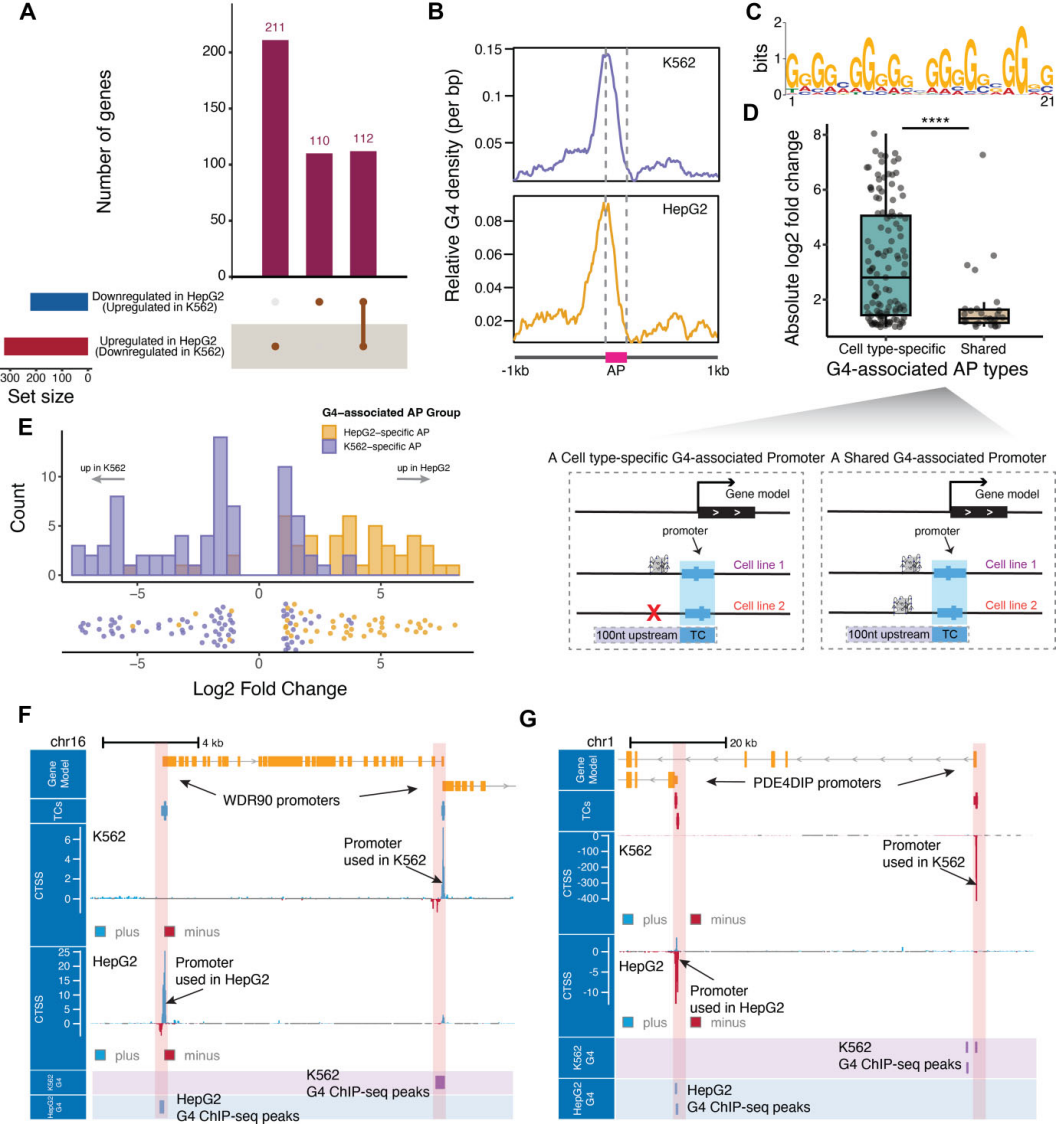
G4 structures are associated with the establishment of APs. (**A**) The number of genes containing alternative promoters derived from comparing the activity of promoters in K562 and HepG2 cell lines based on NET-CAGE data. (**B**) Density of G4 structures centered on APs. Purple (top) and orange (bottom) represent the density of G4s around AP regions in K562 and HepG2 cells, respectively. The vertical axis represents the relative density. The region between the gray dashed lines corresponds to the scaled AP regions. (**C**) The sequence logo of the motif identified in AP regions using the MEME Suite. (**D**) Top: the absolute log2 fold change in activity between cell-type-specific G4-associated alternative promoters and shared G4-associated alternative promoters. ^****^: *P*-value < .0001. Bottom: schematic diagrams of a cell-type-specific G4-associated alternative promoter and a shared G4-associated alternative promoter. A cell-type-specific G4-associated alternative promoter refers to a promoter associated with G4s in both cell lines (K562 and HepG2 cells in this study), while a shared G4-associated alternative promoter refers to promoters that are only associated with G4s in a particular cell line. (**E**) The histogram (top) shows the distribution of log2 fold changes in cell-type-specific G4-associated APs. Orange bars represent the counts of G4-associated APs specific to HepG2 cells, while purple bars represent those specific to K562 cells. The log2 fold change is calculated relative to the HepG2 cell line, where values >0 indicate higher promoter activity in HepG2 cells, and values <0 indicate higher activity in K562 cells. The scatter plot (bottom) displays the individual log2 fold changes of APs. Examples showing the association between alternative promoters and G4 structures in the WDR90 **(F)** and PDE4DIP **(G)** genes. The genomic tracks display the following from top to bottom: gene annotations, TC annotations, CTSS in the K562 and HepG2 cell lines, followed by G4 ChIP-seq peaks in K562 and HepG2 cells (K562 G4 and HepG2 G4). The red and blue CTSS signals indicate transcription initiation events on the positive and negative strands, respectively.

We and others have previously reported a positive correlation between G4 structures and promoter activity [13, 14]. This raises a natural question: can G4 structures also modulate the activity of APs in cells? To investigate this, we collected NET-CAGE data for the K562 and HepG2 cell lines and analyzed differential promoter usage between them (see the “Materials and methods” section). The endogenous G4 landscape of these cell lines has been well characterized by G4 ChIP-seq experiments. In this study, we employed CAGEfightR for the detection and annotation of TCs. TCs that contained G4 ChIP-seq peaks either internally or within 100 bp upstream were defined as G4-associated TCs (see the “Materials and methods” section). We excluded genes that contained only one single expressed promoter, which accounted for the majority of genes (87.7%, Supplementary Fig. S1A). As a result, we identified 573 differentially used APs (FDR < 0.05 and |fold change| > 2), with 341 exhibiting increased promoter activity in HepG2 cells and 232 exhibiting increased activity in K562 cells (Supplementary Fig. S1B). Notably, 26.2% of these promoters were associated with G4 structures (Supplementary Fig. S1C). Compared with background promoters, the proportions of APs associated with G4 structures in neither, one, or both cell lines differed significantly (Fisher’s exact test, *P*-value = 1.52 × 10^−13^; Supplementary Fig. S1D). Promoters lacking G4s were moderately more frequent in APs than in all promoters (ratio = 1.17; Supplementary Fig. S1D), whereas those associated with G4s in only one cell line showed comparable proportions (ratio ≈ 1.0; Supplementary Fig. S1D). In contrast, promoters associated with G4s in both cell lines were substantially less represented among APs (ratio = 0.30; Supplementary Fig. S1D). At the gene level, we observed that 211 genes exclusively contained promoters with increased activity in HepG2 cells, whereas 110 genes exclusively contained promoters with increased activity in K562 cells (Fig. 1A). Additionally, 112 genes contained promoters showing both increased and decreased activities (Fig. 1A).

Disregarding potential long-range enhancer–promoter interactions mediated by G4 structures, we hypothesize that the regulatory influence of G4s on transcriptional initiation primarily operates over short distances. In other words, G4 structures are expected to be located in close proximity to the sites where they exert their regulatory functions. Hence, we examined the distribution pattern of G4 structures around APs. As expected, a significant peak was observed immediately upstream of APs (Fig. 1B), while a distinct G4 motif was detected in the AP region using MEME (Fig. 1C). Moreover, the G4 ChIP-seq signal intensity was significantly higher in APs overlapping G4 peaks than in those without G4 peaks (one-tailed Wilcoxon test, *P*-value = 1.96 × 10^−38^ for K562 and *P*-value = 1.46 × 10^−25^ for HepG2; Supplementary Fig. S1E and F).

Intriguingly, we found that the formation of G4 structures in the two cell lines may influence the differential activity of APs. Specifically, for promoters associated with G4 structures in the K562 cell line, APs exhibited greater differences in G4 signal intensity (ΔG4 = K562 − HepG2; Supplementary Fig. S1G; one-tailed Wilcoxon test, *P*-value = 5.52 × 10^−3^) compared with non-APs. A similar trend was observed for promoters associated with G4 structures in the HepG2 cell line (ΔG4 = HepG2 − K562; Supplementary Fig. S1H; one-tailed Wilcoxon test, *P*-value = 4.19 × 10^−10^). Furthermore, APs associated with G4s in both cell lines (shared G4-associated APs) tend to exhibit reduced fold changes compared to those associated with G4s in only one of the cell lines (cell-type-specific G4-associated APs; Fig. 1D; one-tailed Wilcoxon test, *P*-value = 3.95 × 10^−5^). These results suggest that G4 structures may contribute to cell-type-specific promoter regulation. Moreover, we observed that the presence of G4 structures may play a facilitating role in AP formation. In different cell lines, G4-associated APs exhibit distinct activity biases (Fig. 1E). For instance, HepG2-specific G4-associated APs predominantly exhibit higher activity in HepG2 cells, whereas K562-specific G4-associated APs show higher activity in K562 cells (Fig. 1E). To better understand the directionality of G4-mediated regulation, we then focused on genes showing a clear, single-directional AP usage bias between the two cell lines and categorized them into four representative patterns according to the direction of G4’s effect: “G4 promotes,” “G4 represses,” “G4 bidirectionally promotes,” and “G4 bidirectionally represses.” Genes showing mixed regulatory relationships (i.e. both promoting and repressing effects of G4s within the same gene; *n* = 26) were excluded from this analysis. Most cases indicate a positive regulatory role (84 *promotes*), while repression is also observed (23 *represses*). Three genes showed bidirectional promotion, and none showed bidirectional repression (Supplementary Fig. S2). We hypothesize that the differential formation of G4 structures at specific promoter regions between the two cell lines is a key factor modulating AP formation, as the co-occurrence of G4s may lead to partial offsetting effects. To further illustrate this association, we present two examples from K562 and HepG2 cells. In the WDR90 gene, differentially used promoters correlate with upstream G4 formation, which coincides with promoter activity (Fig. 1F). Likewise, in the PDE4DIP gene, distinct promoters are used in K562 and HepG2 cells, each associated with G4 structures at their respective loci (Fig. 1G). These findings suggest a conserved relationship between G4 formation and promoter selection.

### G4 ligand treatment promotes the formation of alternative promoters

**Figure 2.**
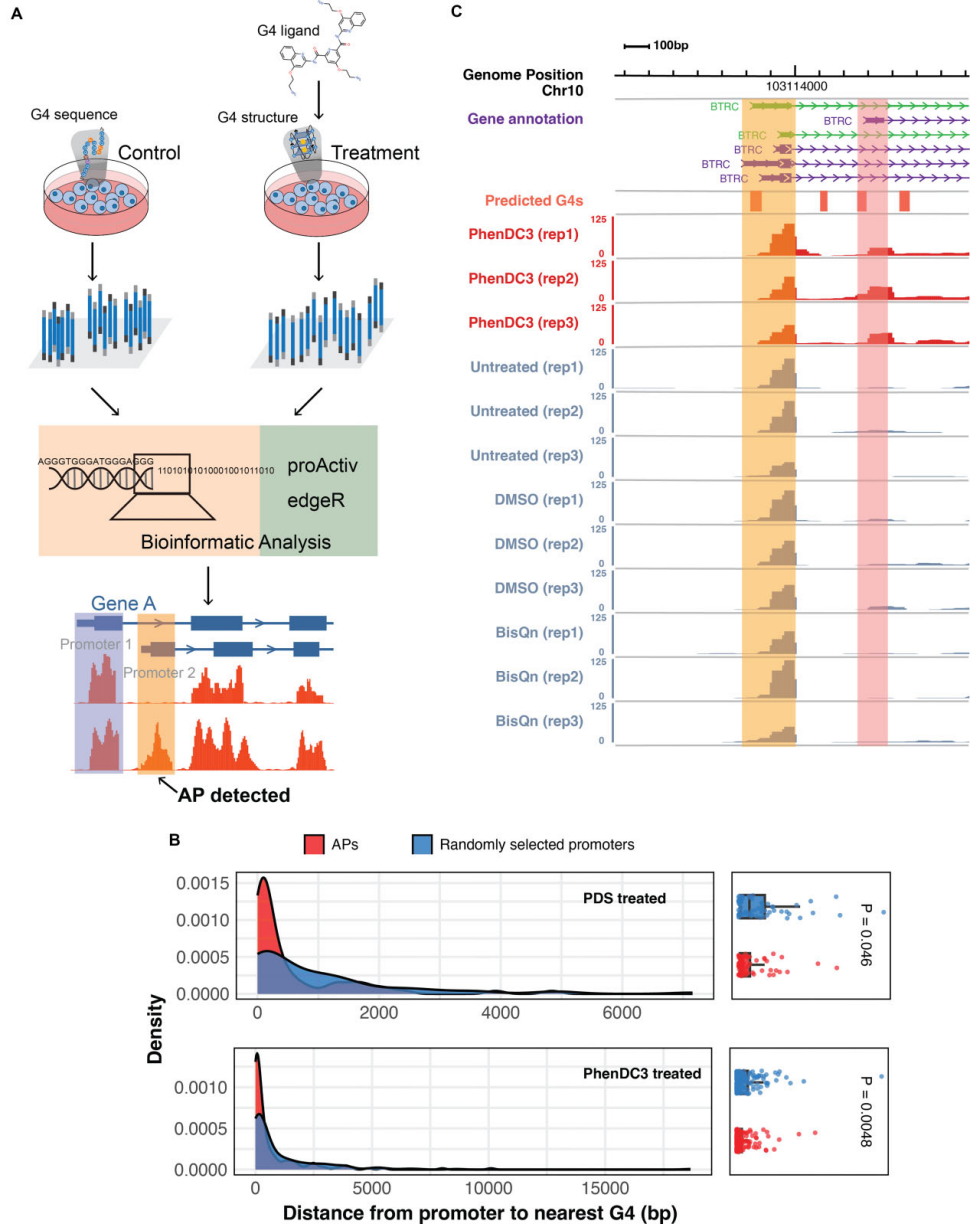
Detection of G4-associated alternative promoters following ligand treatment. (**A**) Principle of our bioinformatic pipeline to detect APs. Briefly, under the treatment of G4 ligands, some of the G4s in cells may transition from a linear sequence state to secondary structures, thereby potentially influencing the usage of promoters. The differential analysis of RNA-seq data between ligand-treated and control groups through bioinformatics software can yield alternative promoters. (**B**) Density plots illustrating the distributions of distances from each alternative promoter (red) or a randomly selected promoter (blue) to its nearest genome-wide predicted G4. The box plots on the right summarize the corresponding distance values for each group. Sample sizes were *N* = 69 for APs and randomly selected promoters in the PDS-treated group (upper panel), and *N*= 157 for those in the PhenDC3-treated group (lower panel). G4s were predicted by G4Hunter, and promoter information was obtained by reanalyzing RNA-seq data from GSE133419. (**C**) The genome browser displays an alternative usage promoter. The top track shows the annotation of the human genome (GENCODE V19 genes). The orange track indicates the positions of G4 sequences predicted by G4Hunter software, while the red and light gray tracks represent RNA-seq data from the PhenDC3-treated and control groups [untreated, DMSO, and bisquinolinium (BisQn)-treated]. Two masks highlight the upstream and downstream promoters of the BTRC gene.

Above genome-wide analysis showed a significant association between endogenous G4 structures and APs. Nevertheless, association is not equivalent to causality. To further explore their connections, we developed a bioinformatics pipeline to investigate whether the stabilization of G4 structures can affect the alternative usage of promoters (Fig. 2A). Our analytical pipeline is based on the following assumption: G4 ligands should stabilize G4 structures. Although some G4-prone sequences are expected to be in a double-stranded form, G4 ligand treatment may promote their folding into stable conformations. Consequently, these ligand-stabilized G4 structures may potentially influence the utilization of promoters in cells, leading to alterations in RNA expression patterns. Then, differential promoter usage patterns can be identified through bioinformatics algorithms by comparing RNA-seq data between the control and ligand-treated groups (Fig. 2A; see the “Materials and methods” section).

We retrieved RNA-seq data for HT1080-ST fibrosarcoma human cells (GSE133419), both treated and untreated with G4 ligands, from publicly available databases and analyzed them using our bioinformatics pipeline to identify APs. As a result, several dozen APs were detected following treatment with PhenDC3 and PDS ligands, but not with PhenDC6, for which minimal changes were observed (Supplementary Fig. S3 and Supplementary Table S1). Notably, the number of promoters showing increased activity was 2–4 times higher than those exhibiting decreased activity (Supplementary Table S1), suggesting that G4 secondary structure formation may contribute to the activation of APs, though this effect may vary depending on the treatment conditions. Furthermore, we found that G4s were closer (smaller mean promoter-G4 distance) to significantly altered promoters than to randomly selected promoters (Fig. 2B and Supplementary Fig. S4), which in turn suggests that G4s may play a role in regulating the activity of these promoters. We speculate that the close proximity of G4s to APs may induce local chromatin topological changes or facilitate the binding of specific transcription factors, thereby influencing the activation of these APs.

As a case, a new promoter that contains two G4Hunter-predicted G4s was activated in the BTRC gene after PhenDC3 treatment (Fig. 2C). BTRC is a gene that mediates IκBα ubiquitination and proteasomal degradation and is involved in diseases such as cancer [25]. This activation suggests that PhenDC3 may influence transcription by promoting the formation of G4 secondary structures in this region, thereby contributing to promoter activation.

## Discussion

**Figure 3.**
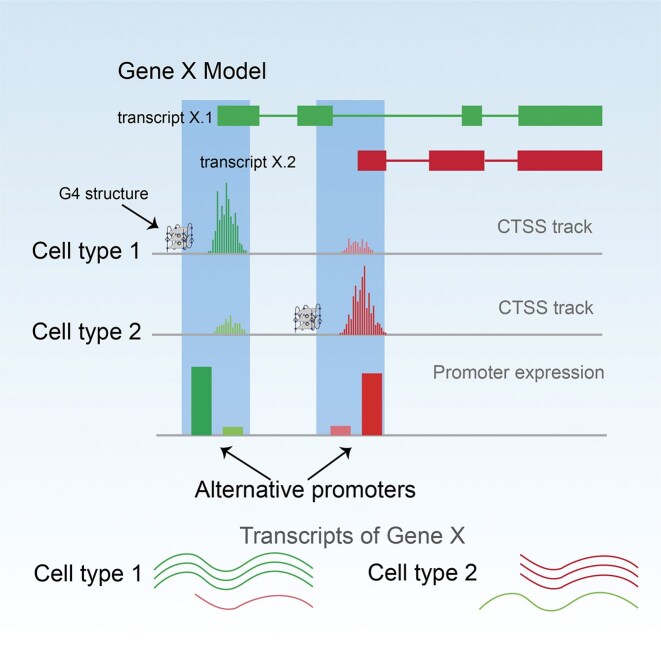
Schematic diagram illustrating the impact of G4 structure formation on the utilization of promoters. The differential formation of G4 structures within the promoter region of a specific gene contributes to the differential utilization of promoters, thereby leading to transcriptome diversity between cell types.

G4s, as noncanonical secondary structures that can regulate various biological functions, have attracted widespread attention. Through bioinformatics analysis, we have shown that G4 structures are associated with the selection of APs, which offers a novel direction for elucidating the mechanisms underlying transcript isoform generation independent of alternative splicing (Fig. 3).

Over the years, researchers have extensively studied the role of G4s in regulating gene expression, spanning from individual genes to the entire genome [14, 26, 27]. However, most G4-related studies have overlooked a fundamental phenomenon: a gene may produce different transcripts, possibly due to the selection of APs. Certainly, as a post-transcriptional process, alternative splicing also contributes to the diversity of transcripts [28–30]. We noticed that a recent study provides a comprehensive overview of the regulatory role of G4s in alternative splicing [31]. Therefore, combined with our findings, we believe that G4 plays a critical role in shaping the diversity of transcripts, both for the regulation of APs and alternative splicing. This is crucial since different transcript isoforms can generate variations in protein-coding sequences, [32], which may consequently affect gene functions and even alter their associations with disease initiation and progression [33–36].

We propose that the regulatory role of G4s in promoter activity is the basis for the formation of APs in certain genes, and that this impact is generally positive from a genome-wide perspective. However, this is not an absolute rule, as we also observed a small subset (approximately 20%; Supplementary Table S2) of genes with reduced promoter activity potentially associated with G4s, in agreement with a previous study [37]. This positive regulatory effect may depend on the recruitment role of G4s on transcription factors [14, 15, 38, 39]. In this regard, G4 structures may cooperate with cell-type-specific transcription factors to regulate promoter activity, which can partly explain the distinct transcriptional profiles observed between different cell lines. Therefore, G4s can be considered as integral components of promoter regulation. In addition, G4 structures can influence the local epigenetic environment to some extent, and this influence, in turn, can modulate transcriptional events. For example, in the chicken DT40 cells with REV1 deficiency, it was found that the occurrence of G4 at the promoter region of BU-1A gene can determine the pattern of H3K4me3 and H3K9/14ac [40]. Another example is that G4 structures can maintain a local low methylation state by sequestering DNMT1 [41], and the removal of methylation is beneficial for activating the expression of genes [42, 43]. In addition, other factors, such as genomic stability or DNA damage and repair [44], may also contribute to differential promoter usage induced by G4s, though direct evidence is lacking. For example, oxidative guanine lesions within G4-prone promoter regions can modulate G4 folding, thereby regulating transcription [45–47]. This might suggest a possible effect on promoter usage. Notably, some helicases and G4-binding proteins, such as XPD and XPB [48], are also involved in the regulation of transcription and nucleotide excision repair. It would be interesting to explore novel G4-based mechanisms that can affect promoter activity, especially in the context of diseases, considering that aberrant promoter activation or silencing may be pivotal in pathogenesis [49, 50].

The differential presence of G4 structures in a specific promoter region under different conditions (e.g. K562 versus HepG2) is an important prerequisite for the formation of APs. We speculate that this is due to a similar “force” exerted by G4 structures on the promoter. For instance, if a promoter is associated with G4 structures in both cell lines, then this promoter is likely to be expressed in these two cell lines, making it ineligible as a candidate AP. Moreover, we observed that the activity fold change of this type of promoter tends to be lower, which could be owing to the mutual counteracting effects of G4s on the differential activity of these promoters in the two cell lines. Therefore, in future studies, it would be important to identify the G4 structures that are differentially used, e.g. those that are formed specifically in promoter regions in cancer cells but not in adjacent normal tissue. Such G4 structures may be linked to transcript abnormalities. These hypotheses will require experimental validation, which is beyond the scope of this study, yet our findings provide a genome-wide framework that should guide future gene-level investigations into G4-associated promoter regulation.

By analyzing the transcriptome data of cells treated with G4 ligands, we further supported the notion that the formation of G4 structures is commonly associated with the generation of APs. Currently, it remains unclear whether G4 ligands affect the transcriptome solely in a G4 structure-dependent manner. Alternatively, G4 ligands may also indirectly influence gene expression through secondary effects on transcriptional regulators or chromatin state. Nevertheless, the stabilization of G4 structures is one of the mechanisms by which G4 ligands impact gene expression. PhenDC3 and PDS are two widely used ligands known for their ability to stabilize G4 structures [51, 52]. In cells treated with G4 ligands, we observed a greater number of APs showing increased activity than those showing decreased activity, which suggests that G4 stabilization tends to positively influence promoter activity [53]. It would also be interesting to consider other naturally occurring G4 ligands, such as heme, since a previous study has demonstrated that porphyrin derivatives can interact with G4 structures and influence transcriptional regulation [54].

We used stringent criteria (absolute fold change > 2 and false discovery rate < 0.05) to identify significantly affected promoters in HT1080-ST cells treated with PDS, PhenDC3, and PhenDC6 ligands. While some APs were observed after PDS and PhenDC3 treatments, only three APs were detected after PhenDC6 treatment under the same threshold. This discrepancy suggests that PhenDC6 induced only subtle transcriptional changes in this cellular environment, insufficient to be corrected by multiple testing with a false discovery rate of 5%. When the threshold was relaxed to an absolute fold change > 1 and *P*-value < .05 (without FDR adjustment), PhenDC6 treatment led to a markedly increased number of APs (Supplementary Table S2). This indicates that although PhenDC6 may indeed induce promoter use alterations detectable at the nominal significance level, these changes do not survive correction for multiple comparisons, reflecting either weaker biological effects or greater variability in cellular response. Future studies with increased sample size may be required to more definitively determine the regulatory impact of PhenDC6 on promoter use. In addition, several other possibilities may exist. For example, different G4 ligands may preferentially stabilize G4s with distinct conformations, which could lead to variations in the number of stabilized G4 structures. Other factors, including the possibility that different ligands may influence cellular activity to varying degrees and thereby lead to differential promoter usage, should also be considered. Taken together, further experimental validation is needed to elucidate how these ligands influence G4 structures and regulate promoter activity.

Understanding the regulation of promoters through long-range interactions [55], such as enhancers, is beyond the scope of this study, although it is a common mechanism for gene expression regulation [55]. Previously, we reported that G4s may serve as mediators for enhancer-driven gene expression modulation [56], which was subsequently supported by another study [57]. Hence, whether G4s can potentially modulate the formation of APs through chromatin higher-order structures is also an intriguing question that remains to be answered.

Collectively, our results establish G4 structures as functional regulatory elements that modulate promoter usage in living cells.

## Supplementary Material

lqaf208_Supplemental_File

## Data Availability

The predicted G4 sequences for human genome (hg19) were stored and can be accessed at https://doi.org/10.5281/zenodo.17743920. Other next-generation data used in this study can be obtained from public databases, as described in the “Materials and methods” section. All analysis scripts are written in R language and executed in Linux system environment (Ubuntu). The scripts have been deposited on Zenodo and can be publicly accessed at https://doi.org/10.5281/zenodo.17743880.

## Notes

Present address: Department of Biology, Stanford University, Stanford, CA 94305, United States
