# Supplementary material for: G-quadruplex structures as modulators of alternative promoter usage
Source: NAR Genom Bioinform. 2025 Dec 31;7(4):lqaf208. doi: 10.1093/nargab/lqaf208 (PMC12754776; doi:10.1093/nargab/lqaf208)
Supplement: lqaf208_Supplemental_File [file lqaf208_supplemental_file.docx]

**G-Quadruplex Structures as Modulators of Alternative Promoter Usage**

Rongxin Zhang^1,2,3*^ & Jean-Louis Mergny^1*^

1 Laboratoire d’Optique et Biosciences (LOB), Ecole Polytechnique, CNRS, INSERM, Institut Polytechnique de Paris, 91120 Palaiseau, France.

2 State Key Laboratory of Digital Medical Engineering, School of Biological Science and Medical Engineering, Southeast University, 211189 Nanjing, China.

3 Current address: Department of Biology, Stanford University, Stanford, CA 94305, USA.

* Authors to whom correspondence should be addressed: [jean-louis.mergny@inserm.fr](mailto:jean-louis.mergny@inserm.fr) or [rongxinzhang@outlook.com](mailto:rongxinzhang@outlook.com)

Supplementary information

**Supplemental Figures**


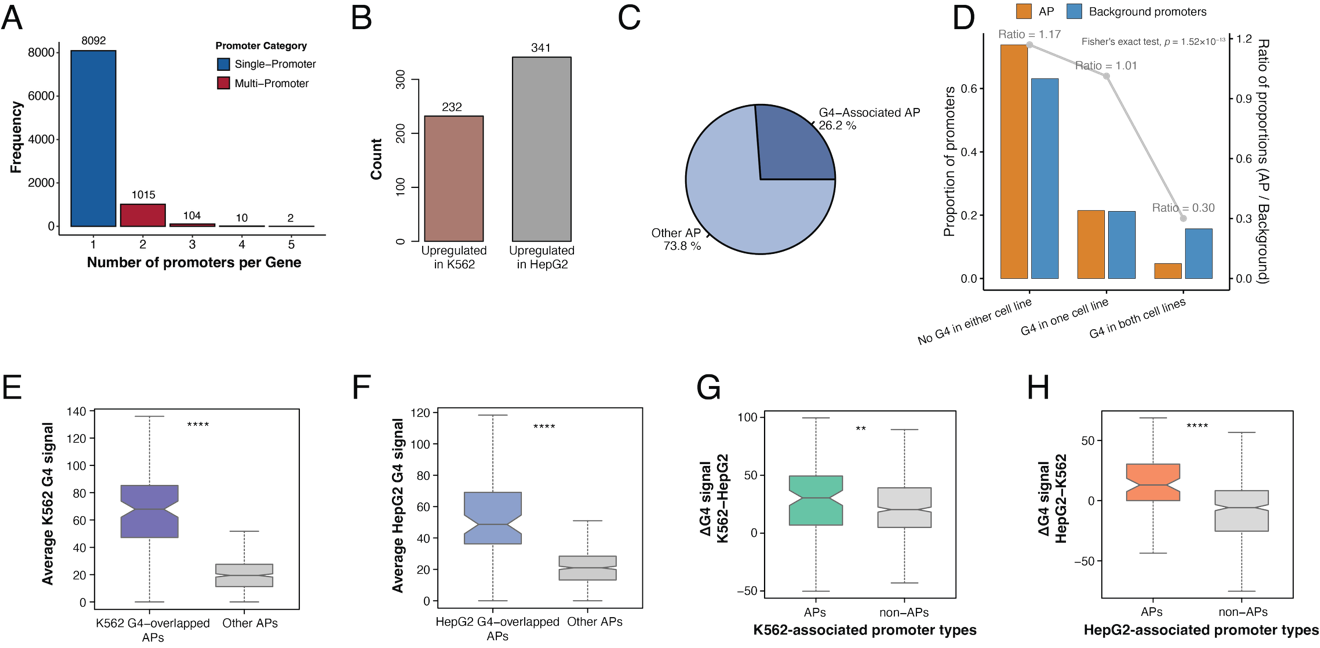


**Figure S1.** (**A**) The frequency of genes with different numbers of expressed promoters. Blue bar represents genes with only one expressed promoter, while red bar represents genes with multiple expressed promoters. (**B**) The number of upregulated alternative promoters in K562 and HepG2 cell lines. (**C**) The proportion of alternative promoters (APs) associated with G4 structures (defined as those containing G4s within 100 bp upstream) or not. (**D**) The proportions of promoters associated with G4 structures in neither, one, or both cell lines are shown for APs and background promoters, respectively. The background group includes all promoters identified in genes with at least two promoters. Bars represent the proportion of promoters within each category, and the gray line indicates the ratio of proportions (AP/background). (**E**) Notched box plot showing the average G4 ChIP-seq signals in K562 cells for K562 G4-associated APs and other APs. (**F**) Same as (**E**), but for HepG2 cells. (**G**) Notched box plot showing the ΔG4 signal (K562 - HepG2) between K562 G4-associated APs and non-APs (promoters with no statistically significant difference in activity). (**H**) The same analysis as in (**G**), but performed for HepG2 cells, showing the ΔG4 signal (HepG2 - K562).


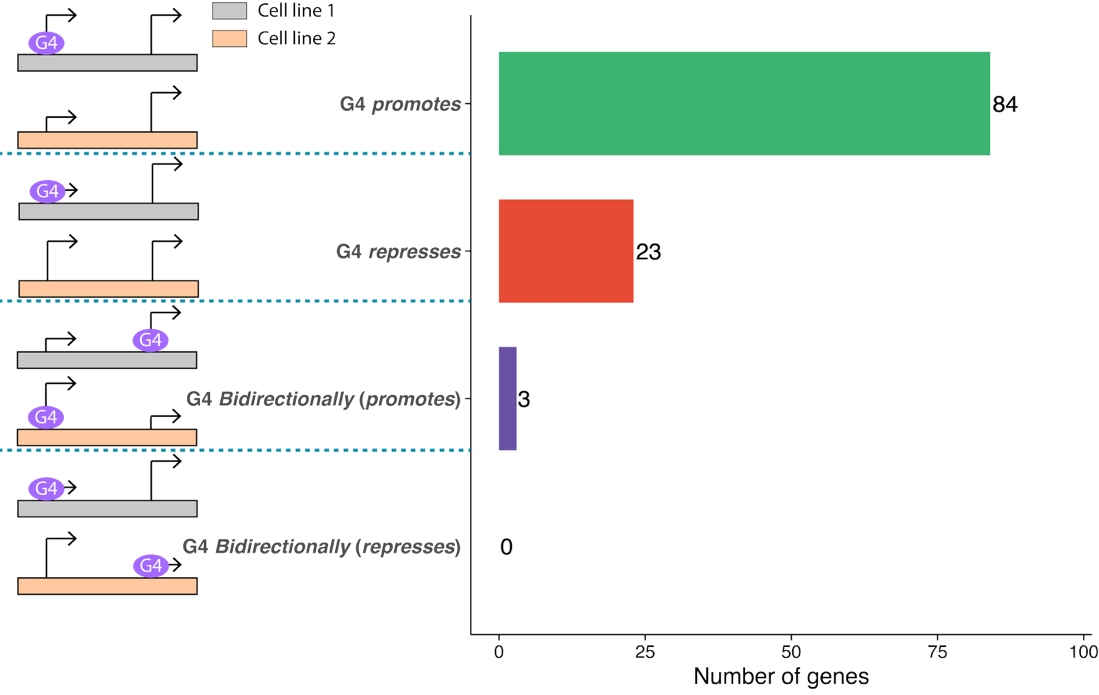


**Figure S2.** Left panel: Schematic illustration of four representative patterns of G4-associated regulation on differentially used APs. The patterns are defined according to the direction of G4-associated effects: “G4 promotes”, “G4 represses”, “G4 bidirectionally (promotes)”, and “G4 bidirectionally (represses)”. Right panel: Bar plot showing the number of genes belonging to each category. Only genes exhibiting clear, single-directional AP usage bias between the two cell lines were included in the analysis.


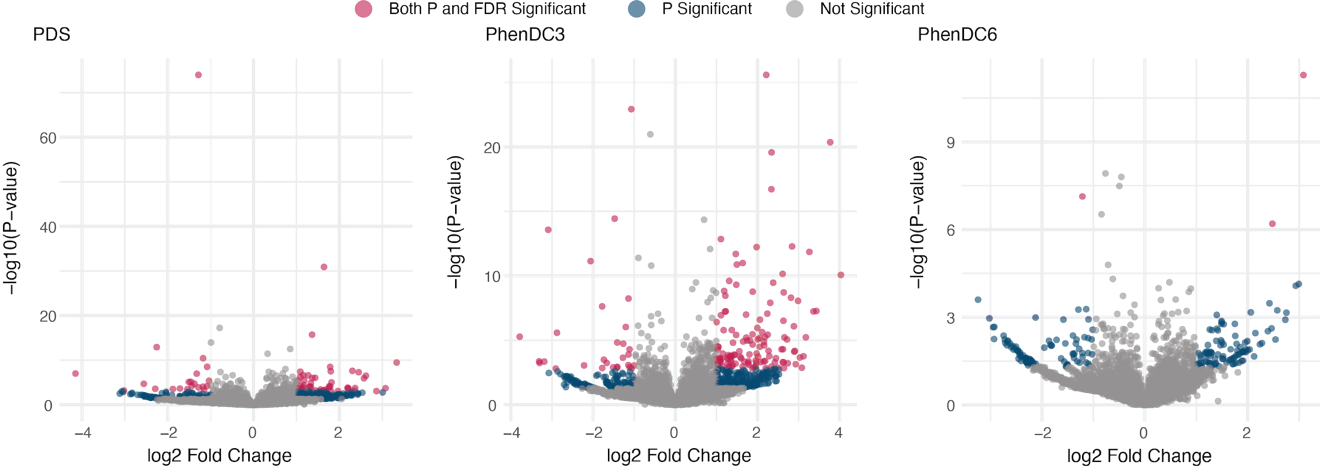


**Figure S3.** Reanalysis of G4 ligand-treated RNA-seq data (GSE133419). From left to right, the volcano plots display the fold change of promoters in the HT1080-ST fibrosarcoma cell line following treatment with PDS, PhenDC3, and PhenDC6, respectively. Red dots indicate promoters with an absolute fold change greater than 2 and a false discovery rate (FDR) below 0.05, while blue dots represent promoters that are statistically significant at p-value < 0.05 but do not pass the FDR significance threshold. In each panel (from left to right), the red dot counts are 69, 157, and 3, and the blue dot counts are 296, 349, and 195, respectively.


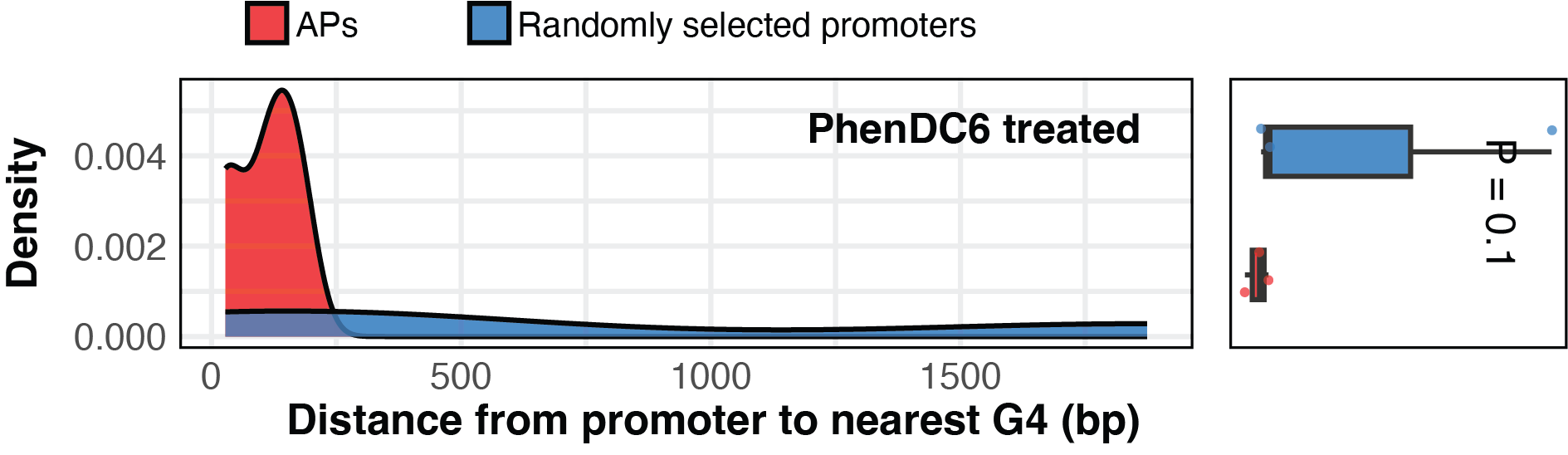


**Figure S4.** The distribution of distances between alternative promoters obtained after PhenDC6 treatment and their nearest G4s (red), while the blue curve represents the distances between randomly selected promoters and their nearest G4s. The box plot on the right displays the individual distance values for both groups. N = 3 for APs and randomly selected promoters in the PhenDC6-treated group. Promoter information was derived from reanalysis of GSE133419 RNA-seq data, and G4s were predicted using G4Hunter.

**Supplemental Tables**

**Table S1.** The count of upregulated and downregulated (|log2fc| > 1, FDR < 0.05) alternative promoters after treatment with different G4 ligands. Results from reanalysis of GSE133419 RNA-seq data.

| **G4 ligand** | **Upregulated** | **Downregulated** | **Total** |
| --- | --- | --- | --- |
| PDS | 48 | 21 | 69 |
| PhenDC3 | 128 | 29 | 157 |
| PhenDC6 | 2 | 1 | 3 |

**Table S2.** The count of nominally upregulated and downregulated (|log2fc| > 1, *p*-value < 0.05) alternative promoters after treatment with different G4 ligands. Results from reanalysis of GSE133419 RNA-seq data.

| **G4 ligand** | **Upregulated** | **Downregulated** | **Total** |
| --- | --- | --- | --- |
| PDS | 247 | 118 | 365 |
| PhenDC3 | 343 | 163 | 506 |
| PhenDC6 | 101 | 97 | 198 |
